# Two may be better than one: PD-1/PD-L1 blockade combination approaches in metastatic breast cancer

**DOI:** 10.1038/s41523-019-0130-x

**Published:** 2019-10-08

**Authors:** David B. Page, Harry Bear, Sangeetha Prabhakaran, Margaret E. Gatti-Mays, Alexandra Thomas, Erin Cobain, Heather McArthur, Justin M. Balko, Sofia R. Gameiro, Rita Nanda, James L. Gulley, Kevin Kalinsky, Julia White, Jennifer Litton, Steven J. Chmura, Mei-Yin Polley, Benjamin Vincent, David W. Cescon, Mary L. Disis, Joseph A. Sparano, Elizabeth A. Mittendorf, Sylvia Adams

**Affiliations:** 1 Providence Cancer Institute; Earle A. Chiles Research Institute, Portland, OR USA; 2 Division of Surgical Oncology and the Massey Cancer Center, Virginia Commonwealth University, Richmond, VA USA; 3 Department of Surgery, Division of Surgery, University of New Mexico; University of New Mexico Comprehensive Cancer Center, Albuquerque, NM USA; 4 Laboratory of Tumor Immunology and Biology, National Cancer Institute, Bethesda, MD USA; 5 Wake Forest University School of Medicine, Winston-Salem, NC USA; 6 University of Michigan, Ann Arbor, MI USA; 7 Cedars Sinai Medical Center, Los Angeles, CA USA; 8 Department of Medicine and Breast Cancer Research Program, Vanderbilt University Medical Center, Nashville, TN USA; 9 The University of Chicago, Chicago, IL USA; 10 Genitourinary Malignancies Branch, National Cancer Institute, National Institutes of Health, Bethesda, MD USA; 11 Columbia University Medical Center, New York, NY USA; 12 Ohio State Wexner Medical Center, Columbus, OH USA; 13 MD Anderson Cancer Center, Houston, TX USA; 14 Mayo Clinic, Rochester, MN USA; 15 University of North Carolina, Chapel Hill, NC USA; 16 Division of Medical Oncology and Hematology, Department of Medicine, Princess Margaret Cancer Centre, University Health Network and University of Toronto, Toronto, ON Canada; 17 University of Washington, Seattle, WA USA; 18 Department of Medical Oncology, Montefiore Medical Center, Albert Einstein Cancer Center, Albert Einstein College of Medicine, Bronx, NY USA; 19 Division of Breast Surgery, Department of Surgery, Brigham and Women’s Hospital; Breast Oncology Program, Dana-Farber/Brigham and Women’s Cancer Center, Boston, MA USA; 20 Perlmutter Cancer Center, NYU School of Medicine, New York, NY USA

**Keywords:** Tumour immunology, Breast cancer

## Abstract

Antibodies blocking programmed death 1 (anti-PD-1) or its ligand (anti-PD-L1) are associated with modest response rates as monotherapy in metastatic breast cancer, but are generally well tolerated and capable of generating dramatic and durable benefit in a minority of patients. Anti-PD-1/L1 antibodies are also safe when administered in combination with a variety of systemic therapies (chemotherapy, targeted therapies), as well as with radiotherapy. We summarize preclinical, translational, and preliminary clinical data in support of combination approaches with anti-PD-1/L1 in metastatic breast cancer, focusing on potential mechanisms of synergy, and considerations for clinical practice and future investigation.

## Introduction

In the spirit of the Hippocratic dictum to “first, do no harm,” a guiding principle in the management of metastatic breast cancer is to favor less treatment rather than more, unless clear evidence of synergy exists.^1^ For example, sequential single-agent chemotherapy is favored over multi-agent chemotherapy because it is better tolerated with similar overall survival (OS).^2^ Recently however, there has been a resurgence of enthusiasm for combination approaches, this time with immune checkpoint antibodies against programmed death 1 (PD-1) or its ligand (PD-L1), based upon preclinical evidence of therapeutic synergy, and recent trials demonstrating acceptable tolerability of these agents with standard-of-care treatment modalities including chemotherapy, radiotherapy, hormone-directed therapies, and targeted therapies (Table 1).^3–18^

**Table 1:** Selected clinical trials demonstrating safety of anti-PD-1/L1 combination therapies

Therapeutic class	Anti-PD-1/L1	Secondary agent	Phase	*n*	Summary	Ref
Chemotherapy	Atezolizumab	Nab-paclitaxel	III	902	“IMpassion130”; Improved OS and ORR in PD-L1 + cancers	^3^
	Pembrolizumab	Paclitaxel, nab-paclitaxel, or gemcitabine/carboplatin	III	858	“Keynote-355”	^4^
	Pembrolizumab	Capecitabine	Ib	14	ORR 43%; 7% grade 3 diarrhea	^5^
	Pembrolizumab	Eribulin	II	104	“ENHANCE-1”; ORR 15% PD-L1+; Grade >3 19.5%	^6^
	Pembrolizumab	Doxorubicin, cyclophosphamide, paclitaxel	III	69	“ISPY-2”; Improved Path CR TNBC and ER+; 7% grade 3	^7^
	Durvalumab	Nab-paclitaxel	II	174	“GeparNuevo”; 48% Path CR; 27% irSAE	^8^
Radiotherapy	Pembrolizumab	Radiotherapy	II	9	33% ORR, no overlapping toxicities	^9^
	Pembrolizumab	Radiotherapy (SBRT)	I	73	13% ORR, 9% grade 3, pre/post biopsies	^10^
CDK4/6i	Pembrolizumab	Abemeciclib	II	28	“JPCE” ORR 14%; Grade >3 11%	^11^
	Avelumab	Palbociclib	II	220	“PACE”	^12^
HER-2-targeted	Pembrolizumab	T-DM1	I	27	NCT03032107	
	Pembrolizumab	Trastuzumab	Ib/II	58	“PANACEA” 15% ORR for PD-L1+ (n = 6/40), 0% ORR PD-L1-, 29% grade 3 + AE	^13^
	Durvalumab	Trastuzumab	I	15	NCT02649686	
	Atezolizumab	T-DM1	II	202	“KATE2” 44% Grade 3+ AE, PFS HR = 0.82 v. T-DM1/placebo (p = NS)	^14^
PARPi	Pembrolizumab	Niraparib	II	55	“TOPACIO” 29% ORR, 49% DCR; 22% BRCAmut	^15^
	Durvalumab	Olaparib	II	30	“MEDIOLA” 90% wk12 DCR, no overlapping toxicities	^16^
HDACi	Atezolizumab	Entinostat	Ib/II	81	Closed to accrual late 2018	^17^
IDOi	Pembrolizumab	Epacadostat	I/II	39	“ECHO-202”; ORR 10% TNBC	^18^

*AE* adverse event, *CDK4/6i* cyclin-dependent kinase 4 and 6 inhibitors, *ER*+ estrogen receptor-positive, *HDACi* histone deacetylase inhibitors, *HER2* human epidermal growth factor receptor 2, *HR* hazard ratio, *N* number, *ORR* overall response rate, *CR* complete response, *OS* overall survival, *PARPi* poly(ADP ribose) polymerase inhibitors, *PD-1* programmed death 1, *PD-L1*+ programmed death ligand 1-positive, *PD-L1*- programmed death ligand 1-negative, *PFS* progression-free survival, *NS* not significant, *DCR* disease control rate, *BRCAmut* germline BRCA gene mutated, *T-DM1* trastuzumab emtansine, *TNBC* triple-negative breast cancer, *wk* week, *IDOi* IDO inhibitors

Cytotoxic chemotherapy has pleiotropic immunomodulatory effects that may synergize with anti-PD-1/L1. Recently, the first randomized anti-PD-1/L1 combination trial in metastatic breast cancer, IMpassion130, provided proof-of-concept that anti-PD-1/L1 plus chemotherapy can be safe and more effective than chemotherapy alone. In the trial, atezolizumab (anti-PD-L1) prolonged progression-free survival (PFS) in combination with first-line nab-paclitaxel (7.2 versus 5.5 months, HR 0.80, 95% CI: 0.69–0.92) in the entire population, with a preliminary analysis suggesting prolonged OS in the 41% of subjects with tumors containing at least 1% PD-L1-positive immune cells (25.0 versus 15.5 months, HR 0.62, 95% CI: 0.45–0.86).^3^ In the second interim analysis, OS was prolonged for the PD-L1-positive population (25.0 versus 18.0 months, HR 0.71, 95% CI: 0.54–0.93) but not the overall population (21.0 versus 18.7 months, HR 0.86, 95% CI: 0.72–1.02, *p* = 0.077).^19^ The combination was generally well tolerated without compromising health-related quality of life as reported by patients,^20^ thereby reducing concerns of harm and increasing enthusiasm for investigation of other anti-PD1/L1 combinations. In addition, the robust negative predictive value of the integral PD-L1 biomarker (SP142 antibody) was promising, allowing for future selection of individuals most likely to derive benefit. Numerous randomized phase III studies combining anti-PD-1/L1 with standard-of-care therapies are ongoing and will be reported over the next several years, potentially increasing the breadth of options for combination immunotherapy in breast cancer.^21^

However, given the perils of cross-trial comparison, one foreseeable clinical challenge is to ascertain the relative efficacy of dozens of feasible anti-PD-1/L1 combination approaches in metastatic breast cancer. The goals of this review are to describe immunologic mechanisms of synergy of various standard therapeutic approaches with anti-PD-1/L1, summarize available preclinical data, and discuss clinical use and future investigations of anti-PD-1/L1 combination approaches in metastatic breast cancer.

## Cytotoxic chemotherapy

Cytotoxic chemotherapy remains a standard-of-care for metastatic breast cancer, with commonly employed agents including microtubule-targeting agents (paclitaxel, nab-paclitaxel, eribulin, docetaxel), anthracyclines (epirubicin, doxorubicin), anti-metabolites (capecitabine, gemcitabine), alkylating agents (cyclophosphamide), and platinums (cisplatin, carboplatin). The immunomodulatory effects of chemotherapy have been the subject of extensive review,^22^ and include expansion or activation of effector cell populations (including natural killer [NK] cells, dendritic cells [DC], and T cells), depletion or inhibition of suppressor cell populations (tumor-associated macrophages [TAM], myeloid derived suppressor cells [MDSC], Tregs), and induction of immunogenic cell death (ICD), a stress response associated with release of danger-associated molecular patterns (DAMPs) signals and enhanced antigen presentation.^22–27^ Chemotherapy is also associated with interferon gamma secretion and adaptive PD-L1 upregulation.^28,29^ For all these reasons, there has been significant interest in evaluating the efficacy of combining chemotherapy with anti-PD-1/L1. On the other hand, patients who have been extensively pretreated with cytotoxic therapy seem less likely to respond to immunotherapy, suggesting immunosuppressive mechanisms may dominate in the context of more extensive therapy.

While there are preclinical models demonstrating the efficacy of anti-PD-1/L1 plus various chemotherapy agents,^29,30^ there are fewer data comparing the relative efficacy of the various chemotherapy agents plus anti-PD-1/L1, and results across animal models are inconsistent. For example, cyclophosphamide-containing regimens were among the most effective potentiators of anti-PD-1/L1 response in one study,^30^ whereas cyclophosphamide plus anthracycline failed to enhance anti-PD-1/L1 response in another.^31^ Because immune effects of chemotherapy are varied, it becomes difficult to compare the effects on the basis of pharmacodynamic activity alone. For example, anti-metabolites (5-FU and gemcitabine) may be superior to anthracycline or cyclophosphamide in depleting MDSCs, doxorubicin may be superior in inducing ICD, whereas cyclophosphamide may be superior in depleting Tregs.

In the phase III IMpassion130 trial, atezolizumab improved PFS when added to first-line nab-paclitaxel in metastatic triple-negative breast cancer (TNBC) in the entire study population, but OS was only prolonged in patients with tumors bearing PD-L1-positive immune cells.^3^ These results led to regulatory approval in the first-line setting for PD-L1-positive disease. However, an earlier phase I study demonstrated a response rate of 24% and durable responses in the first-line setting with single-agent atezolizumab,^32^ raising the possibility that the benefits observed with the addition of atezolizumab are additive rather than synergistic. Ongoing studies will address whether benefits of the taxane/atezolizumab combination can be seen in patients with a shorter disease-free interval than 12 months as studied in IMpassion130 and whether alternative chemotherapy backbones could offer similar or greater clinical benefit. The IMpassion131 study is similar to IMpassion130, however evaluating atezolizumab plus paclitaxel rather than nab-paclitaxel (NCT03125902). Importantly, the results of this study may provide clarity on whether prophylactic steroids impair clinical benefit to anti-PD-L1. The multi-arm, non-comparative phase II “TONIC” trial evaluated various induction chemotherapy regimens or radiation followed by anti-PD-1 (nivolumab) in metastatic TNBC. In this small study, highest objective responses were observed following induction cisplatin (23% ORR) and induction doxorubicin (35% ORR), however these findings must be confirmed in a larger study.^33^ The optimal sequencing of anti-PD-1/L1 with other therapies remains a topic of considerable debate. The KEYNOTE-355 phase III trial will provide additional randomized data of pembrolizumab versus various chemotherapy backbones (NCT02819518). Of note, this trial uses a different PD-L1 IHC assay (DAKO 22c3 antibody) for patient selection, which recently was found to classify more TNBCs as PD-L1-positive, compared to the SP142 assay.^34^ The impact of PD-L1-discordance may require additional investigation.

## Radiotherapy

In the metastatic setting, ionizing radiotherapy is frequently employed to palliate symptoms (for example, to bone metastases or chest wall lesions) or to delay progression of central nervous system metastases using either stereotactic radiosurgery or whole brain radiotherapy. The principal mechanism of radiotherapy is to induce lethal DNA damage to tumor cells or tumor-associated stroma. However, radiotherapy can enhance anti-tumor immunity by engaging both innate and adaptive responses. In some cases, radiotherapy may be associated with regression of non-irradiated tumors, coined the “abscopal effect.” Radiation-induced DNA damage may lead to cell death and serve as a source of antigen and danger signals that facilitate DC maturation and cross-presentation of tumor antigens to prime tumor-specific T cell responses.^35,36^ However, it has been shown that the vaccine-effect of radiotherapy is modest, and that synergy with checkpoint blockade may depend on pre-existing immunity.^37^ Similar to chemotherapy, radiotherapy is associated with release of DAMPs such as uric acid, high mobility group box 1 (HMGB1), calreticulin, and double stranded DNA, which act as immunologic adjuvants to activate myeloid cells and facilitate subsequent chemokine release and T-cell recruitment. Radiotherapy may also upregulate MHC class I and FAS adhesion molecules, which may counteract adaptive loss of MHC or beta 2 microglobulin.^38^ Conversely, radiotherapy can cause immunosuppressive effects, including upregulation of the PD1/PDL1 axis, upregulation of suppressive macrophage receptors including Mertk,^39^ expansion of Tregs, and possibly apoptosis of tumor infiltrating lymphocytes (TILs).

In preclinical models, suppressive effects of radiotherapy can be mitigated in combination with anti-PD-1/L1. In a melanoma model, anti-CTLA4 plus radiotherapy was associated with PD-L1 upregulation, and the addition of anti-PD-L1 reversed T-cell exhaustion, promoted clonal T-cell expansion within the tumor, and enhanced response.^40^ It is difficult to ascertain the optimal dose and schedule of radiation plus immune checkpoint inhibitor. Increased dose is associated with more profound release of DAMPs including ATP and HMGB1, but may also promote immunosuppressive effects such as induction of exonucleases that eliminate cytosolic DNA, a key messenger of DC activation and downstream T-cell priming.^41,42^ In a comparison of various fractionation schedules plus anti-PD-1 using MOC1 and MC38 murine models, higher-dose hypofractionated radiotherapy (8 Gy x 2) was superior to low-dose fractionated radiotherapy (2 Gy× 10) in controlling tumor, enhancing interferon production, and upregulating PD-L1.^43^ In a breast cancer model, hypofractionated (8 Gy × 3) was superior to high single dose therapy (20–30 Gy).^41^ Radiation may also cause systemic lymphopenia (with fractionated radiotherapy causing more profound lymphopenia compared to hypofractionated),^44^ and conversely, systemic immunosuppression may influence efficacy.^45^ The timing of radiation may also influence response, with one study showing concurrent therapy superior to sequential.^46^ Radiation combined with anti-PD-1/L1 has been well tolerated in patients with metastatic breast cancer with preliminary reports of tumor response in lesions outside the radiation field.^47^ Optimizing radiotherapy dose and timing will likely be the subject of future clinical trials. Furthermore, other immune stimulatory agents such as toll-like receptor 3 agonists and fms related tyrosine kinase 3 ligand (Flt3L), may synergize with radiotherapy and may hold unique promise in conjunction with anti-PD-1/L1.^48^

## Endocrine therapy

Estrogen/progesterone modulation remains a cornerstone of palliative therapy of hormone receptor (HR)-positive metastatic breast cancer. FDA-approved estrogen-directed therapies include a selective estrogen receptor modulator (tamoxifen), aromatase inhibitors (exemestane, letrozole, and anastrozole), and a selective estrogen receptor degrader (fulvestrant). These agents may be used as monotherapy (with or without ovarian suppression), or in combination with targeted agents such as mammalian target of rapamycin (mTOR) inhibitors (everolimus) or cyclin-dependent kinase 4/6 (CDK4/6) inhibitors. Most HR-positive breast cancers and about half of TNBCs express the androgen receptor (AR) to some degree, prompting emerging interest in evaluating AR inhibition as a therapeutic strategy.^49^ Androgen signaling is known to play a negative regulatory role in central (thymic) T-cell production, and androgen ablation/blockade has been shown to facilitate increases in thymus size, lymphocyte count, thymic recombination of the T-cell receptor, and T-cell cytolytic activity.^50^ In murine breast cancer models, androgen blockade was associated with enhanced T-cell killing via upregulation of the apoptosis ligand, TRAIL.^51^ In prostate cancer models, AR blockade increased immune responses to vaccination.^52^ Finally, in a prostate cancer trial, pembrolizumab plus enzalutamide was associated with increased tumor and DC PD-L1 expression, increased circulating PD-1-positive T-cells, and clinical response following enzalutamide progression.^53,54^ Anti-PD-1/L1 agents combined with androgen blockade are currently being evaluated across a number of clinical trials in the metastatic breast cancer setting (NCT03650894, NCT02971761). Combinations with anti-estrogens are also ongoing, including the multi-arm MORPHEUS trial that combines fulvestrant with atezolizumab +/− other targeted approaches (NCT03280563).

## Cyclin-dependent kinase 4/6 inhibitors

Cyclin dependent kinase 4 and 6 inhibitors (CDK4/6i) have dramatically changed the treatment of metastatic HR-positive breast cancer. There are three FDA-approved agents: palbociclib, ribociclib, and abemaciclib. CDK4/6i are thought to work primarily by inducing cytostasis via G1 cell-cycle arrest, but have also been shown to induce apoptosis in vitro.^55^ Preclinical evidence suggests that CDK4/6i promote anti-tumor immunity by increasing antigen processing and presentation. CDK4/6i also activate tumor cell expression of endogenous retroviral elements and stimulate interferon signaling, resulting in enhanced tumor antigen presentation.^56,57^ In human epidermal growth factor receptor 2 (HER2)-positive breast cancers, CDK4/6i also increase expression of multiple antigen processing and presentation genes, including MHC Class I and Class II.^56^ They may also modulate NK cell activity.^58^ Teo and colleagues observed increased expression of cell-surface calreticulin in TNBC cell lines (HCC1806 and MDA-MB-231) after treatment with ribociclib, suggesting that CDK4/6i can induce ICD.^59^ In addition, CDK4/6i augment T cell effector function while markedly suppressing proliferation of regulatory T cells. As cell cycle inhibitors, CDK4/6i decrease T cell proliferation; however, CDK4/6i increase the activation of effector T cells and modulate gene expression.^57,60^ Preclinical and clinical studies have confirmed increased tumor infiltrating T cells^61^ and decreased Tregs within treated tumors.^56,57,60,61^

Given their place in standard treatment, a favorable side effect profile, and the documented beneficial immune effects, CDK4/6i may be a promising agent to combine with anti-PD-1/L1. CDK4/6i increase PD-L1 expression in vivo, with mounting preclinical data suggesting synergy with PD-1/PD-L1 blockade.^57,59,60,62^ For example, in a CT26 model, the clinical activity of abemaciclib was dependent on immunity, and combination anti-PD-L1 plus abemaciclib resulted in superior disease control with complete responses. Of note, concurrent therapy was superior to sequential therapy in this model. A phase Ib study of pembrolizumab plus abemaciclib in heavily pretreated patients with PD-L1-positive estrogen receptor-positive/HER2-negative advanced cancer showed an acceptable safety profile and clinical activity (overall response rate [ORR] 14.3% at 16 weeks with a 75% disease control rate)^63^ compared to historical controls for single agent pembrolizumab (ORR 12%)^64,65^ or single agent abemaciclib (ORR 20% with a 42% disease control rate).^66^

## HER2-directed therapy

Overexpression of HER2 is observed in ~20% of breast carcinomas and is associated with an aggressive phenotype. The standard-of-care first-line therapy for metastatic HER2-positive breast cancer is systemic therapy with taxane plus dual anti-HER2 antibody therapy (trastuzumab and pertuzumab), which is associated with impressive gains in OS, and survival correlates with the degree of TILs.^67^ Both trastuzumab and pertuzumab are capable of eliciting antibody-dependent cellular cytotoxicity (ADCC) via interactions of the antibody fragment crystallizable region (Fc) with Fc receptors found on NK cells and macrophages.^68^ Trastuzumab emtansine (T-DM1) is an antibody-drug conjugate, approved in the second-line trastuzumab-resistant setting, that augments the cytotoxic effect of trastuzumab via conjugation with the DM1 chemotherapy moiety. DM1 induces DC maturation and stimulates anti-tumor immunity.^69^ In murine models, T-DM1 therapy is associated with robust increases in T-cell infiltration, Th1 polarization, PD-1/PD-L1 expression, and intratumoral Tregs infiltration. Combination anti-PD-1 plus anti-CTLA-4 plus T-DM-1 was superior to T-DM-1 or anti-PD-1/CTLA-4 in a preclinical model.^69^ Lapatinib is an oral targeted inhibitor of EGFR and HER2, approved in combination with capecitabine or trastuzumab for metastatic HER2-positive breast cancer. Because lapatinib stabilizes HER2 protein at the cell membrane, it may also enhance the ADCC-effect of trastuzumab.^70^ Chemotherapeutic agents including taxanes may also enhance trastuzumab-mediated ADCC.^71^ Additional agents, including margetuximab, are being developed to maximize the ADCC-mediated immunotherapeutic effect of HER2-targeted therapy.^72^

In addition to modulating ADCC, anti-HER2 antibodies may also interact with adaptive immune responses.^73^ In a murine model, the activity of anti-HER-2 was dependent on cytotoxic T-cells and interferon secretion, and was improved in combination with anti-PD-1.^74^ One additional consideration for HER2-positive breast cancer is the antigenic potential of the HER2 protein. The E75 peptide vaccine, derived from an immunodominant epitope of the HER2 extracellular domain, has been shown to induce E75-specific cytotoxic T-cell responses in humans, and is being evaluated for clinical efficacy in the adjuvant setting in a phase III clinical trial.^75,76^ Trastuzumab was shown to facilitate DC uptake and antigen presentation of HER2, and increase E75-specific T-cell responses.^75^ HER2 signaling is also associated with downstream activation of the PI3K/mTOR/AKT pathway; therefore blockade may have secondary immune effects including PD-L1 upregulation. However, analysis of the TCGA database found no significant correlation between the mRNA expression levels of HER2 and PD-L1 in 790 available cases of breast cancer.^77^

In a phase Ib/II trial, a 20% ORR was observed with pembrolizumab plus trastuzumab in trastuzumab-resistant PD-L1-positive tumors.^13^ In a similar trial, durvalumab plus trastuzumab was safe at standard full doses, but no responses were observed in a heavily pre-treated population.^78^ Ongoing clinical trials will evaluate whether combination therapy with anti-PD-1/L1 is effective in earlier lines of therapy, including a first-line trial evaluating standard-of-care first-line taxane/trastuzumab/pertuzumab +/− atezolizumab (NCT03199885). Of note, in the randomized phase II KATE2 study, the addition of atezolizumab to second-line T-DM1 only improved PFS, but only in the PD-L1-positive cohort.^14^

## PI3K/AKT/mTOR pathway inhibitors

A recent analysis of 13,349 genomic profiles identified an association of tumor mutational burden with common breast cancer oncogenic driver mutations, including mutations of both the PI3K/AKT/mTOR and RAS/MAPK pathways.^79^ The mTOR inhibitor, everolimus, is approved in metastatic HR-positive/HER2-negative breast cancer in combination with exemestane,^80^ and also improves PFS when added to fulvestrant.^81^ Recently, the PI3K inhibitor, alpelisib, was approved in combination with fulvestrant for tumors bearing an activating PIK3CA mutation.^82^ Inhibitors of AKT are being developed and show promise in clinical trials. Recent studies have implicated this pathway in promoting an immunosuppressive tumor microenvironment^83^ via two mechanisms: (1) increased expression of immunosuppressive cytokines and chemokines which promote recruitment of MDSCs and Tregs^84^ and (2) constitutive expression of PD-L1.^85^ However, the association with PTEN loss/PIK3CA activation and PD-L1 expression was not observed in a small set of human TNBC specimens.^86^ Several pre-clinical studies have suggested that inhibition of the PI3K/AKT/mTOR pathway may decrease Tregs and promote CD8+ memory T-cell differentiation.^87–89^ Preclinical models evaluating the utility of combination therapy are limited, but the addition of anti-PD-1 was found to enhance the benefit of dual blockade of PI3K and CDK4/6 in an AT3OVA in vivo model.^59^ The PI3K-γ isoform has been specifically implicated in the function of TAM, and inhibitors of PI3K-γ are being clinically evaluated in combination with atezolizumab in TNBC (NCT03961698).

## Poly(ADP ribose) polymerase inhibitors

Phase III clinical trials have demonstrated efficacy of PARP inhibitors (PARPi) in metastatic breast cancer patients with a germline BRCA1/2 pathogenic variants (gBRCA). In the Olympiad study, olaparib was associated with an improvement in PFS relative to physician’s choice of non-platinum chemotherapy, showing a median PFS of 7.0 months vs. 4.2 months (HR 0.58, *P* < 0.001), however, OS was not prolonged.^90,91^ Similarly the EMBRACA trial evaluated talazoparib in a similar cohort of patients with a median improvement in PFS of 8.6 months vs. 5.6 months (HR 0.54, *P* < 0.001).^92^ On the basis of these results, olaparib and talazoparib are now FDA-approved for gBRCA metastatic breast cancer.

In addition to direct antitumor effects, PARP inhibition may have immunomodulatory properties that improve or impair therapeutic efficacy in breast cancer. PARP inhibition has been associated with an increased number and effector function of cytotoxic T cells and NK cells, showing synergy with CTLA-4 inhibition in an immunocompetent BRCA1−/− model of ovarian cancer, with efficacy driven by improved peritoneal T cell effector function and IFNγ production with combination therapy.^93^ Treatment of human BRCA−/− UWB1.289 cells with IFNγ caused significantly greater cytotoxicity when the cells were treated with a PARP inhibitor,^93^ suggesting PARP inhibition may prime cells for IFNγ mediated cell death. Recently, PARP inhibition by olaparib was found to induce robust innate and adaptive immune responses in a *BRCA*-deficient murine ovarian cancer model, as well as enhanced benefit in combination with anti-PD-1, via cytosolic DNA sensing and activation of the stimulator of interferon genes (STING) pathway.^94,95^ Conversely, PARPi may also interfere with healthy immune function. PARP is known to interact with and activate NF-κB, a master regulator of innate immune function.^96^ PARP is necessary for optimal DC differentiation, activation, and stimulation of T cells.^97^ PARP deficiency has been attributed to increases in frequency and function of Tregs, decreased Th1 cytokine/chemokine function, deficiencies in Th2 differentiation, deficiencies in B-cell antibody class switching and somatic immunoglobulin hypermutation.^98,99^ Given the anticipated pleotropic effects of PARPi on anti-tumor immunity, more mechanistic studies in the context of ongoing clinical trials as well as randomized trials (such as NCT02849496) will be required to evaluate the synergistic potential of PARP inhibition in combination with anti-PD-1/L1. Recently the phase II single arm MEDIOLA trial evaluated olaparib in combination with durvalumab in patients with gBRCA, and demonstrated a disease control rate at 12 weeks of 80%.^100^

In breast cancer, PARPi have been shown to increase tumor cell expression of PD-L1, thereby suppressing the antitumor T cell response, but also to have a synergistic effect when given with PD-1 inhibition.^101^ This effect may be due at least in part to inhibition of PARP-mediated CD8+ T cell apoptosis driven by reactive oxygen species produced by tumor cells.^102^ The Topacio/Keynote-162 trial evaluated niraparib and pembrolizumab in a phase II single arm trial with an ORR of 28% and disease control rate of 50%, with the best responses being demonstrated in patients with a tumor *BRCA* mutation.^15^

## Emerging therapeutic modalities

Epigenetic modifying agents, including histone deacetylase inhibitors (HDACi), are undergoing phase III evaluation in metastatic breast cancer and may be immunomodulatory.^103,104^ HDACi target epigenetic pathways inducing transcription modifications associated with growth inhibition, apoptosis, cell differentiation and ultimately anti-tumor effects.^105^ MDSCs which can suppress T-cell responses, pose an important limitation to immune therapy for breast cancer, but may also serve as a potential target for amplifying host immunity. This has been shown in animal models and in patients with breast cancer.^104,106^ Preclinical work demonstrates that HDACi may reduce the activity of MDSCs and Tregs,^104,107^ upregulate MHCI/II, increase sensitivity of breast cancer cells to cytotoxic T-cell mediated lysis, direct NK cell-mediated lysis, and facilitate ADCC.^108^ Exploratory analyses from the phase II clinical trial ENCORE 301 (exemestane +/− entinostat) demonstrated an increase in HLA-DR-positive monocytes and a decrease in granulocytic and monocytic MDSCs in patients treated with HDACi.^109^ Immunomodulatory activity was correlated with histone acetylation of peripheral mononuclear cells (suggested biomarker of response) and clinical benefit. Given the immunomodulatory effects of HDACi, it is not surprising that multiple preclinical studies have found synergy with the combination of HDACi and checkpoint blockade in breast cancer and other solid tumors.^104,110,111^ DNA methyltransferase inhibitors (DNMTi, e.g., azacitidine, decitabine, guadecitabine) and various systemic chemotherapies (gemcitabine, doxorubicin, and others) also increase MHCI and tumor antigen and reduce systemic and intratumoral MDSCs, potentially augmenting anti-PD-1/L1.^104^

Targeted inhibition of the oncogenic RAS-MAPK pathway, a driver of some breast cancers, may also have immunostimulatory effects. Genomic or transcriptomic activation of the RAS-MAPK pathway has been associated with decreased TIL infiltration in residual disease specimens of patients with TNBC treated with neoadjuvant chemotherapy.^112^ RAS-MAPK pathway activity has been shown to suppress antigen presentation by decreasing expression of MHC-I and MHC-II. Furthermore, MEK inhibition has been demonstrated to upregulate MHC and PD-L1 expression, suggesting that combining MEK inhibitor plus anti-PD-1/L1 may be a promising therapeutic strategy. Indeed, this combination has yielded preclinical anti-tumor activity and is now being explored in phase I/II clinical trials. However, additional pre-clinical studies suggest that while MEK inhibition may augment TIL infiltration in TNBC, it may also have the unintended consequence of encumbering T cell proliferation, but may extend the survival and fitness of antigen-specific T-cells in the microenvironment.^113^ MEK signaling occurs downstream of T cell receptor activation. Therefore, inhibition of MEK may also decrease T cell proliferation and cytokine production, which can be overcome by co-administration of T-cell agonists such as anti-OX40.^113^

Additional immunotherapeutic agents, including agents targeting immune-metabolic pathways (adenosine and indoleamine 2,3-dioxygenase 1 [IDO1]) or T-cell agonists (OX40) are being evaluated in conjunction with anti-PD-1/L1 in breast cancer. Adenosine mediates the pro-tumor effects of the ectoenzyme CD73, which is expressed in TNBC and associated with chemotherapy resistance.^114^ Activation of adenosine receptors (A2A-R or A2B-R) suppresses T-cell proliferation, cytokine production, and cytotoxicity.^115,116^ In 4T1 TNBC mouse models, A2A/B inhibition plus anti-PD-l was superior to monotherapy, with the observed benefit dependent on interferon secretion, NK-cells, and CD8+ T-cells.^117^ The adenosine receptor inhibitor, CPI-44, has been evaluated in conjunction with atezolizumab in early clinical trials, but has not been specifically evaluated in breast cancer patients. IDO1 is induced in DCs and macrophages at sites of inflammation, and degrades tryptophan into immune-suppressive metabolites that are associated with T-cell apoptosis, reduced activation, and Treg phenotype differentiation.^118^ In 4T1 TNBC orthotopic mouse models, IDO1 knockout results in reduced lung metastasis and improved survival.^119^ The IDO1 inhibitor, epacadostat, was well tolerated when combined with pembrolizumab, and was associated with a 10% ORR in a small TNBC patient cohort.^120^ Recently, however, epacadostat failed to improve PFS when combined with pembrolizumab in stage IV melanoma.^121^ Finally, OX40 is a T-cell agonist molecule which, when stimulated, may reduce the threshold required for initial T-cell activation. In both MMTV-PyMT and 4T1 mammary carcinoma models, combination anti-OX40 plus anti-PD-1/L1 was associated with improved tumor control, but the synergistic effect was demonstrated only when anti-OX40 was administered in sequence with anti-PD-1/L1. Concurrent therapy was more toxic to mice and associated with surges in both Th1 and Th2 cytokines, highlighting the possibility that compensatory feedback mechanisms could modulate efficacy of combination immunotherapy.^122^

## Discussion

**Fig. 1:**
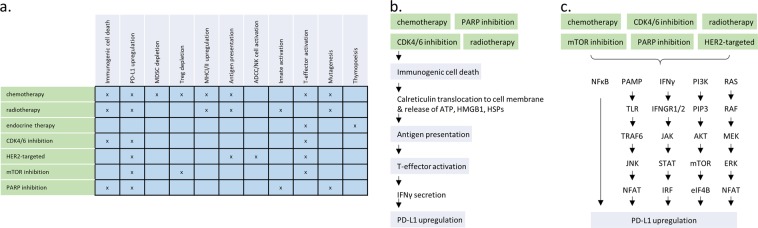
Potential Mechanisms of synergy of anti-PD-1/L1 combination therapies. **a** Standard-of-care therapies in the metastatic breast cancer setting exhibit varied and overlapping immunomodulatory effects that may promote therapeutic synergy with anti-PD-1/L1; **b** Immunogenic cell death is conserved across a number of anti-neoplastic modalities. A hallmark biologic feature is calreticulin exposure from the endoplasmic reticulum, resulting in downstream antigen presentation, T-cell activation, and adaptive PD-L1 upregulation; **c** Another common mechanism of synergy is PD-L1 upregulation, which can occur via modulation of a variety of molecular pathways. PD-L1: programmed death ligand 1; MDSC: myeloid derived suppressor cell; Treg: T-regulatory cell; MHC: major histocompatibility complex; ADCC: antibody-dependent cellular cytotoxicity; NK: natural killer; CDK4/6: cyclin-dependent kinase 4/6; HER2: human epidermal growth factor receptor 2; mTOR: mammalian target of rapamycin; PARP; poly(ADP ribose) polymerase; ATP: adenosine triphosphate; HMGD1: high mobility group box 1; HSPs: heat shock proteins; IFNγ: interferon gamma; NFκB: nuclear factor kappa-light-chain enhancer of activated B-cells; PAMP: pathogen-associated molecular pattern; TLR: toll like receptor; TRAF6: TNF receptor associated factor 6; JNK: c-Jun N-terminal kinase; NFAT: nuclear factor of activated T-cells; IFNGR1/2: interferon gamma receptor 1/2; STAT: signal transducer and activator of transcription protein; IRF: interferon regulatory factor; PI3K: phosphoinositide 3-kinase; PIP3: phosphatidylinositol (3,4,5)-triphosphate; AKT: protein kinase B; eIF4B: eukaryotic translation initiation factor 4B

Pre-clinical, translational, and early clinical data support ongoing efforts to combine anti-PD-1/L1 with standard-of-care and emerging therapies including chemotherapy, radiotherapy, endocrine therapy, and targeted therapy. A number of putative mechanisms of synergy have been demonstrated, some of which are shared across therapeutic modalities (Fig. 1). An emerging clinical challenge is to determine the optimal combination strategy in the face of a wealth of preclinical and clinical data, as well as to determine whether single-agent anti-PD-1/L1 could be effective in a subset of breast cancers. Summarized below are key considerations in the use of anti-PD-1/L1 combination approaches for metastatic TNBC, HR-positive breast cancer, and HER2-positive breast cancer.

In metastatic PD-L1-positive TNBC, the PFS benefit (and preliminary OS benefit) in IMpassion130 provides level I evidence supporting atezolizumab (anti-PD-L1) plus nab-paclitaxel as a standard approach for first-line therapy for patients with a >12 month distant recurrence free interval and PD-L1-positivity. There are insufficient data to guide whether anti-PD-1/L1 can be effectively combined with alternative chemotherapy regimens. Ongoing randomized trials (including Keynote-355, NCT02819518) are addressing this question. In phase I/II studies of anti-PD-1/L1 monotherapy, efficacy diminishes substantially in later lines of therapy, suggesting that earlier treatment may be more effective. Biomarker assessments from ongoing trials may guide future personalization of chemotherapy plus anti-PD-1/L1 according to patient and/or tumor characteristics. Subjects with PD-L1-negative tumors did not benefit from the addition of atezolizumab, and therefore should be considered for clinical trials evaluating anti-PD-1/L1 in combination with novel agents. A number of existing therapies can induce PD-L1 upregulation, and may be promising for study in the PD-L1-negative TNBC population.

For subjects with HR-positive metastatic breast cancer, tumors are less likely to be PD-L1-positive.^64^ Several combination strategies have mechanistic basis, including anti-PD-1/L1 plus CDK4/6i (with or without aromatase inhibitor), chemotherapy, mTOR inhibition, HDACi, DNMTi, AR blockade, or radiotherapy. With further research, novel biomarkers including high-throughput genomic/genetic profiling and advanced histologic approaches may be developed to personalize therapy.

Subjects with HER2-positive breast cancer benefit from a multitude of approaches. An ongoing phase III randomized trial will evaluate first-line pertuzumab/trastuzumab/paclitaxel +/− atezolizumab (NCT03199885). An additional combination to be considered is T-DM1 plus anti-PD-1/L1, which in a phase II trial was associated with improved PFS but only in PD-L1-positive tumors (NCT02924883). Combinations with novel agents, such as HER2-directed vaccines, are promising and warrant clinical evaluation.

Despite the relative safety of anti-PD-1/L1 combination therapies, the potential for long-term toxicity exists. A prominent example is immune-related endocrinopathy (thyroid or adrenal dysfunction), which has been observed with anti-PD-1/L1 combination therapy and may require lifelong hormone replacement therapy. Resources should be devoted to evaluate patient reported outcomes and extend the time period for such measures to be assessed. Furthermore, novel phase I statistical designs should be employed to capture late toxicities in dose decision-making. For example, the time-to-event continual reassessment method starts with a target dose limiting toxicity (DLT) rate that the investigators deem acceptable, and the first patient is followed for DLT.^123^ The toxicity information of previously treated patients is adaptively combined with new patient data using a Bayesian approach, allowing for continuous reassessment of toxicity estimates. Owing to the allowance of staggered enrollment without the need for accrual suspension during DLT follow-up, this design has the potential to substantially shorten the trial duration compared to traditional phase I designs. Furthermore, it has been shown that this design assigns a greater proportion of patients to the target dose.

## Conclusion

The IMpassion130 clinical trial serves as proof-of-principle that anti-PD-1/L1 combination approaches can be safe and effective in metastatic breast cancer. A vast body of preclinical, translational, and clinical data supports ongoing efforts to evaluate a variety of combination approaches. As additional clinical trials are completed and various combination approaches are found to be beneficial, careful evaluation must be made to select the optimal combination strategy given unique patient and tumor characteristics. Moreover, robust, systematic, and streamlined biomarker studies are critical if immunotherapy combination strategies are to become applicable for the majority of breast cancer patients.

## Data Availability

No new datasets were generated or analyzed for this report.
